# Predictors of grit among medical students: a study at a Malaysian Public University

**DOI:** 10.1186/s12909-024-05798-0

**Published:** 2024-07-23

**Authors:** Nurfauzani Ibrahim, Mariam Mohamad, Suraya Abdul-Razak, Mohamed-Syarif Mohamed-Yassin, Noorhida Baharudin

**Affiliations:** 1 Jelebu Health Clinic, Kuala Klawang, Jelebu, 71600 Negeri Sembilan Malaysia; 2 Department of Primary Care Medicine, Faculty of Medicine, Universiti Teknologi MARA, Jalan Hospital, Sungai Buloh Campus, Sungai Buloh, 47000 Selangor Malaysia; 3 Institute of Pathology, Laboratory and Forensic Medicine (I-PPerForM), Universiti Teknologi MARA, Jalan Hospital, Sungai Buloh Campus, Sungai Buloh, 47000 Selangor Malaysia; 4 Department of Public Health Medicine, Faculty of Medicine, Universiti Teknologi MARA, Jalan Hospital, Sungai Buloh Campus, Sungai Buloh, 47000 Selangor Malaysia; 5 Cardio Vascular and Lungs Research Institute (CaVaLRI), Hospital Al-Sultan Abdullah Universiti Teknologi MARA, Jalan Hospital, Sungai Buloh, 47000 Selangor Malaysia

**Keywords:** Grit, Medical students, Education, Personality, Malaysia

## Abstract

**Background:**

Previous literature has demonstrated associations between grit and positive educational and psychological outcomes, such as higher academic grades, lower attrition rates in medical training, and protection from burnout. However, the predictors of grit have yet to be studied, particularly among medical students in Malaysia. This study aimed to determine the level of grit and its predictors among Malaysian medical students.

**Methods:**

A cross-sectional study was conducted among 123 medical students from a public university in Malaysia. Data on sociodemographic and educational characteristics were collected. The student’s personality traits were determined using the Big Five Inventory (BFI), while grit was assessed using the validated 7-item Short Grit Scale (Grit-S). Grit was expressed as a mean score, ranging from 1 (not at all gritty) to 5 (extremely gritty). Multiple linear regression was used to determine the association between the predictors (personality, sociodemographic and educational characteristics) and grit among these students.

**Results:**

The mean grit score was 3.43 (SD 0.57). Based on the multiple linear regression analysis, the grit score was significantly predicted by three personality traits which were extraversion, b = 0.2 (95% CI: 0.07–0.32), agreeableness, b = 0.28 (95% CI: 0.12–0.44) and conscientiousness, b = 0.6 (95% CI: 0.42–0.77). A 1-point increase in the mean extraversion, agreeableness, and conscientiousness scores would independently increase these students’ mean grit scores by 0.2, 0.28, and 0.6, respectively. The sociodemographic and educational characteristics did not significantly predict grit among Malaysian medical students.

**Conclusions:**

The mean grit score among Malaysian medical students is comparable to other medical students in Asia. Extraversion, agreeableness, and conscientiousness personality traits were associated with higher grit. As grit is a dynamic trait, appropriate interventions should be implemented to foster and increase it among these students.

## Background

Intelligence quotient (IQ) measures cognitive ability and has long been a popular predictor of academic achievement [1]. Terman and Oden, in their prospective cohort study, however, discovered that highly accomplished men had only five points higher IQ than the least accomplished ones [2]. They reported that these accomplished men, who were professors, lawyers, or doctors, shared non-cognitive attributes such as perseverance and self-confidence, which predict their achievements more than their IQ [2].

Among many non-cognitive traits, grit is associated with various positive outcomes, such as higher educational achievements and retention in training [3]. The concept of grit was initially introduced by Professor Angela Lee Duckworth in 2007. It is defined as “perseverance and passion for long-term goals, working strenuously toward challenges, and maintaining effort and interest over the years despite failure, adversity, and plateaus in progress” (3, pp1087-1088). Duckworth et al. developed the questionnaire to measure grit, encompassing two domains: consistency of interests (COI) and perseverance of effort (POE). The COI domain described persistency of interest over time, while the POE domain emphasised long-term effort and stamina. Those who exhibit a higher level of grit will not only finish their present tasks but maintain their interest and effort over the years. Grit is essential for students who wish to pursue medical education, as the journey is long and arduous. The training can be compared to a marathon. It requires stamina, which enables students to persevere, maintain their endurance, and eventually reach their dreams of becoming a doctor [3].

The association between grit and positive educational outcomes have been well established [3–5]. In their work, Duckworth et al. studied the effect of grit in several cohorts of students, such as the psychology students in the United States [3]. This study discovered that higher grade point averages (GPA) were associated with increased grit scores [3]. In her study in the United States, Chang found that college student’s academic performance was positively predicted by the scores from the perseverance of effort subscale of grit [6]. These findings were also observed among medical students, where those who had higher grit scores ranked higher in their class and performed better in medical school [4].

In addition to positive educational outcomes, grit has also been discovered to protect medical students from burnout [7]. In the United Kingdom, Maher et al. showed that approximately 5.7% of medical students could not finish medical school, primarily due to academic difficulties and psychological problems [8]. Students who exhibit a higher level of grit would be more likely to complete their medical school despite facing many challenges, as previous literature has shown that grit predicts retention and persistence across various life events, such as graduating from school [9].

The benefit of grit extends beyond undergraduate education. Medical training requires more than a decade of intense learning, from medical students graduating from medical schools to doctors pursuing postgraduate qualifications to become medical specialists. Malaysia, a developing nation, still needs more doctors, particularly medical specialists. The number of general doctors is about 61 158 [10], yet the number of specialist physicians or surgeons is only 13 874 [11]. Grit is an important personal trait for those pursuing long-term goals [3]. Thus, any intervention enhancing grit among medical students would be beneficial in enabling them to complete medical school, attain postgraduate qualifications, and subsequently increase the number of much-needed medical specialists in Malaysia.

Grit can be improved through internal and external factors [12]. The internal factors (student factors) include encouraging and supporting students to pursue their interests. Encouragement from parents and teachers provides ongoing stimulation, essential to fostering grit, while the positive feedback makes them feel fulfilled, content, and happy [12]. Another way to cultivate grit is by allowing students to practice their skills consistently until they become competent [12]. The external factors are the environment. Students were more likely to display a high level of grit when surrounded by people with similar qualities [12]; therefore, role-modelling by colleagues, lecturers, and medical doctors is vital to fostering this positive attribute among medical students.

Various factors have been shown to predict grit. These include sociodemographic factors, such as older age and female gender, and some educational characteristics, such as a higher cumulative grade point average (CGPA) [3, 13]. Additionally, many studies have demonstrated the association between grit and positive non-cognitive attributes, such as conscientiousness personality, self-esteem, and empathy [13, 14]. Internationally, the role of grit in predicting positive educational outcomes among medical students has been previously demonstrated [5, 8]. However, the literature on grit among medical students in Malaysia still needs to be explored. Thus, this study aimed to determine grit and its association with sociodemographic, educational characteristics, and personality traits among undergraduate medical students in a public university in Malaysia.

## Methods

### Study design and population

**Fig. 1:**
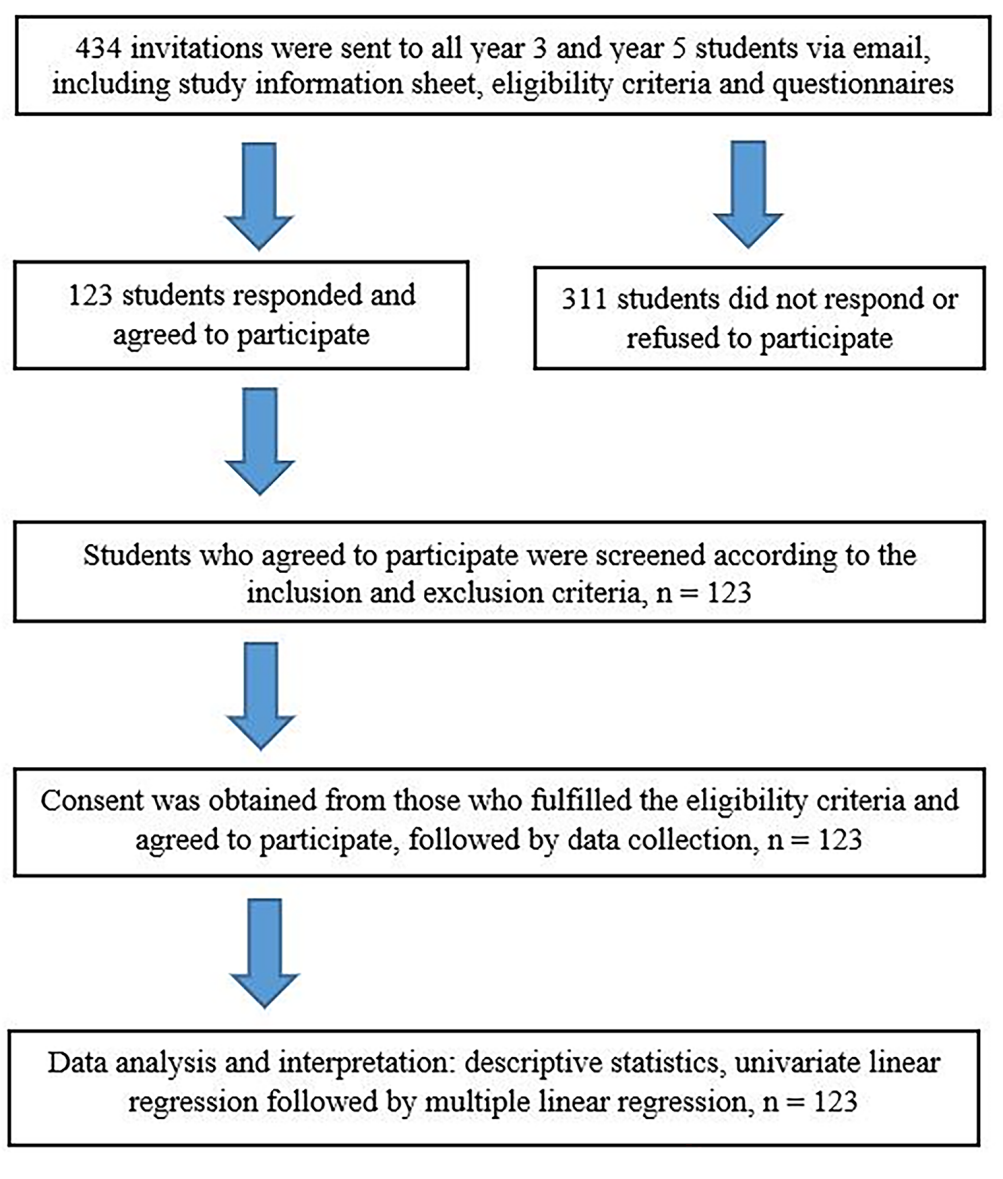
Flowchart of study

This cross-sectional study was conducted among undergraduate medical students in a public university in Selangor, Malaysia. The conduct of the study is outlined in Fig. 1. The inclusion criteria were third and fifth-year undergraduate medical students in the academic year of 2019/2020, aged 18 years old and older. The students from years three and five were chosen because they were in the clinical years of training, which had different curricula and learning methods from the pre-clinical years. Year 4 students were not invited to this study as they participated in the validation of the Short Grit Scale (Grit-S) [15]. The exclusion criteria were: (i) The pre-clinical year students (years 1 and 2), (ii) Year 4 undergraduate medical students in the 2019/2020 academic year who were involved in the validation of the Grit-S, (iii) Students who were attending and receiving treatment for mental illness, which may impair their ability to score the grit questionnaire accurately, or (iv) Students who were missing from class for one month or longer due to any medical illness.

### Study tools

The questionnaire had three sections. The first section included questions about the students’ sociodemographic and educational characteristics. The other sections included the Grit-S and the Big Five Inventory (BFI).

### Sociodemographic and educational characteristics: variable definition

The students’ ethnicity was grouped as Malay or indigenous. Most Malaysians are of Malay ethnicity. The indigenous refers to the native ethnicities other than the Malays, locally known as *“orang asli”*, which includes indigenous minorities from East Malaysia, such as Iban and Kadazan. The household income was categorised according to the Department of Statistics, Malaysia (DOSM). Based on this, household income was grouped into three categories. The categories were: (i) bottom 40% (B40): household income less than Ringgit Malaysia (MYR) 4850 per month, (ii) middle 40% (M40): household income between MYR 4850 to MYR 10,959 and (iii) top 20% (T20): household income more than MYR 10,959 [16]. Based on the DOSM, urban was defined as gazetted areas with a combined population of 10,000 or more [17]. The participants’ town or district of origin was classified based on this definition. English language competency was based on the student’s Malaysian University English Test (MUET) achievements. The MUET categorised English competency into six bands: Band 6 - very good user, Band 5 - good user, Band 4 - competent user, Band 3 - modest user, Band 2 - limited user and Band 1 - extremely limited user.

### Short grit scale (Grit-S)

The study tool is Grit-S, which has undergone adaptation, validation (content, face and construct), and reliability assessments among medical students in Universiti Teknologi MARA (UiTM), Malaysia [15]. For construct validity, exploratory factor analysis revealed a 7-item Grit-S, framed within two domains, which were (i) Consistency of interest (COI) and (ii) Perseverance of effort (POE). Four items were in the COI domain (1, 2, 4, and 5) and three (3, 6, and 7) in the POE domain. The internal consistency for COI and POE domains were 0.73 and 0.75, respectively. The adapted Grit-S had an overall Cronbach’s alpha of 0.73 and an intra-class correlation for all items that ranged from 0.62 to 0.80. The values indicated that the adapted Grit-S was reliable and stable over time.

The response for each item was based on a 5-point Likert scale, ranging from ‘1 = not like me at all’ to ‘5 = very much like me’. Items in the COI domains (1, 2, 4, and 5) were reverse-coded as suggested by the Grit-S developer to ensure a consistent scoring method across all items [18]. The total score (7 to 35) was divided by the number of items (seven) to give the final grit score. The mean grit score ranged from 1 (not at all gritty) to 5 (extremely gritty) [18]. Permission to use the original and validated questionnaire was obtained from the questionnaire developer.

### Big five inventory (BFI)

The personality traits were measured using the BFI [19]. This questionnaire consisted of 44 items covering five personality traits, which were extraversion (8 items), agreeableness (9 items), conscientiousness (9 items), neuroticism (8 items) and openness (10 items) [19]. Extraversion describes sociability, talkativeness and expressiveness. Agreeableness includes attributes such as kindness, empathy and cooperation. Conscientiousness is defined by thoughtfulness, goal-oriented behaviours, and organisation, while neuroticism is characterised by moodiness and emotional instability. Openness includes openness to trying new things and being adventurous and creative [20]. Each item of the BFI is scored on a 5-point Likert scale ranging from “1 = strongly disagree” to “5 = strongly agree”, corresponding to how accurately each statement applied to the participants [19]. Some of the items in each domain were negatively phrased and thus needed to be reverse-coded accordingly, as recommended by the questionnaire’s developer [19]. The total score for each personality domain was divided by the number of items in the domain, giving the mean score for each personality trait. The score ranges from one to five, with higher values corresponding to higher levels of that specific personality trait. The BFI has been widely used and demonstrated adequate reliability and validity among Malaysian university students [21, 22]. The subscales for BFI had Cronbach’s alpha values ranging from 0.72 to 0.78 [23].

### Sample size determination

The calculated sample size was 97. It was determined using a sample size formula and calculator for estimating a single mean [24, 25]. The critical value (z) for a 95% confidence interval was 1.96 [26]. The 10% (0.1) precision was deemed acceptable for this study. The attrition rate was estimated based on an earlier study among medical students at the same university, which showed an attrition rate of 10% [15]. In the present study, the data was collected online; thus, the attrition rate was expected to be higher at 20%. Previous research conducted among pharmacy students showed a mean Grit-S score of 3.7 with a standard deviation of 0.5 [27]. Based on a precision of 10% (0.1) and a standard deviation of 0.5, the calculated sample size was 97. The study planned to approach approximately 122 students after considering a 20% attrition rate.

### Student recruitment, sampling method and data collection

Given the Coronavirus Disease 2019 (COVID-19) restrictions where classes were conducted via online platforms, this study’s data collection was done similarly. An invitation email with the Google form link was sent to all year three and year five students. The reminders were sent weekly for one month. The students were categorised as non-responders if they did not respond to the online survey after one month. The data was collected between August and September 2020. The Google forms contained the study information sheet and consent form. The students who were eligible and agreed to participate were recruited. The Grit-S and BFI were self-administered. The students were assigned a unique research identification number to maintain their confidentiality.

### Data assessment and statistical analysis

Data analyses were conducted using the Statistical Package for the Social Sciences Version 23 (IBM SPSS Statistics for Windows, Armonk, NY: IBM Corp, 2016). Continuous variables were expressed as mean with standard deviation (SD) or median with interquartile range (IQR) based on the normality testing. Categorical variables were described in numbers and percentages. The overall grit score and the scores for each domain of the Grit-S (POE and COI) were reported as mean with SD, consistent with the method suggested by its developers [18].

The simple linear regression was performed initially to determine the factors associated with grit. The variables with a p-value of less than 0.25 were included in the multiple linear regressions (MLR) to adjust for the confounders. The threshold of less than 0.25 was chosen to explore the best regression model, as a value of less than 0.05 could potentially fail to identify important variables [28]. From the MLR, a *p*-value of less than 0.05 was considered significant. The results were presented as regression coefficients with 95% confidence intervals (CI). The personality traits, sociodemographics, and educational characteristics were the independent variables. The grit score was the dependent variable.

## Results

### The sociodemographic and educational characteristics of the participants

Of the 434 year three and year five undergraduate medical students, 123 responded to the questionnaires, corresponding to a 28.3% response rate. The respondents had a median age of 23 (IQR 2). Among the 123 respondents, 92 (74.8%) were females. 64 students (52%) were third year and 59 (48%) were fifth-year medical students. Most participants were Malays (95.9%) and spoke Malay as their primary language (98.4%).

As for educational characteristics, most of the students attended non-boarding secondary schools (58.5%), **s**cored more than nine subjects with an A grade for the Malaysian high school education certificate (65%), locally known as “*Sijil Pelajaran Malaysia*” (SPM) examination, and achieved the perfect CGPA of 4.0 in their pre-university preparation course (86.2%). About half (51.2%) were in the bottom 40% of household income. The sociodemographic and educational characteristics of the participants are shown in Table 1.

**Table 1:** Sociodemographic and educational characteristics of the participants (*n* = 123)

Variables	Frequency, *n* (%)
**Age (Years)**	
Median (IQR)	23.00 (2)
**Year of Study**	
3	64 (52)
5	59 (48)
**Gender**	
Male	31 (25.2)
Female	92 (74.8)
**Race**	
Malay	118 (95.9)
Indigenous	5 (4.1)
**Main Language Spoken at Home**	
Malay	121 (98.4)
Other (English/Iban)	2 (1.6)
**Type of Secondary School**	
Non-boarding	72 (58.5)
Boarding	51 (41.5)
**Number of A grades in the Malaysian High School Education Certificate Exam**	
Median (IQR)	9.0 (1)
≤ 8	43 (35)
≥ 9	80 (65)
**CGPA for Preuniversity Course**	
4	106 (86.2)
< 4.0	17 (13.8)
**Type of Preuniversity Course**	
Foundation in Science/Matriculation	113 (91.9)
Diploma in Health Science/Microbiology	10 (8.1)
**Malaysian University English Test (MUET) Band**	
3	32 (26)
4	80 (65)
5	11 (8.9)
**English Grade in the Malaysian High School Education Certificate Exam**	
A	97 (78.9)
B	26 (21.1)
**Household Income Category**	
Bottom 40% (< MYR4850)	63 (51.2)
Middle 40% (MYR4850 – MYR 10,959)	42 (34.1)
Top 20% (> MYR10,959)	18 (14.6)
**Father’s Education Level**	
No formal education/Primary Level	5 (4.1)
Secondary Level	41 (33.3)
Tertiary Level	76 (61.8)
Missing	1 (0.8)
**Mother’s Education Level**	
No formal education/Primary Level	9 (7.3)
Secondary Level	55 (44.7)
Tertiary Level	59 (48)
**Father’s Occupation**	
Managers and professionals	48 (39)
Technicians and support services	38 (30.9)
Pensioner/Unemployed	28 (22.8)
Missing	9 (7.3)
**Mother’s Occupation**	
Managers and professionals	41 (33.3)
Technicians and support services	16 (13)
Pensioner/Housewife	63 (51.2)
Missing	3 (2.4)
**Number of Siblings**	
1 - 2	15 (12.2)
3 - 4	56 (45.5)
≥ 5	52 (42.3)
**Locality of Origin**	
Rural	23 (18.7)
Urban	100 (81.3)

### Personality traits

In this study, Cronbach’s alpha for extraversion, agreeableness, conscientiousness, neuroticism, and openness personality traits was 0.78, 0.71, 0.77, 0.85, and 0.58, respectively. The mean (SD) scores for personality traits in this cohort ranged from 3.01 (0.6) (extraversion) to 3.86 (0.5) (agreeableness). The mean score for each personality trait is presented in Table 2.

### The grit scores

The mean (SD) grit score was 3.43 (0.57), and the mean (SD) scores for the COI and POE subscales were 3.23 (0.74) and 3.69 (0.68), respectively (Table 2).

**Table 2:** The personality traits and grit scores among year three and year five medical students (*n* = 123)

		Mean (SD)	Min	Max
**Personality**				
	Extraversion	3.01 (0.6)	1.63	4.38
	Agreeableness	3.86 (0.5)	2.78	5.0
	Conscientiousness	3.43 (0.45)	1.89	4.56
	Neuroticism	3.11 (0.72)	1.5	4.88
	Openness	3.35 (0.42)	2.3	4.3
**Grit score**		3.43 (0.57)	1.71	4.43
	Perseverance of Effort (POE)	3.69 (0.68)	2.0	5.0
	Consistency of Interest (COI)	3.23 (0.74)	1.25	4.5

### Predictors of grit

Table 3 shows the results from the simple linear regression analysis. From the 18 variables analysed, six (race, extraversion, agreeableness, conscientiousness, neuroticism, and openness personality) had *p*-values of less than 0.25. These predictors were included in the multiple regression analysis.

**Table 3:** Simple linear regression on the predictors of grit

Variables	b	t (df)	*p*-value
Age (years)	0.00	0.04 (121)	0.97
Gender			
Male	Ref		
Female	0.03	0.22 (121)	0.83
Race			
Indigenous	Ref		
Malay	-0.30	-1.17 (121)	**0.24**
Primary Language Spoken at Home			
Other (English/Iban)	Ref		
Malay	-0.08	-0.19 (121)	0.85
Type of Secondary School			
Non-boarding	Ref		
Boarding school	-0.01	-0.01 (121)	0.90
Number of A grade in Malaysian High School Education Certificate Exam	0.02	0.47 (121)	0.64
CGPA for Preuniversity Course			
< 4	Ref		
4	0.01	0.04 (121)	0.97
Type of Preuniversity Course			
Foundation in Science/Matriculation	Ref		
Diploma in Health Science/Microbiology	-0.06	-0.31 (121)	0.76
MUET Band	-0.01	-0.14 (121)	0.88
SPM English Grade			
A	Ref		
B	0.14	1.15 (121)	0.25
Household Income Category (MYR per month)			
Bottom 40% (< MYR 4850)	Ref		
Middle 40%/Top 20% (≥ MYR 4850)	-0.02	-0.16 (121)	0.87
Father Education Level			
No formal education/Primary Level/Secondary	Ref		
Tertiary Level	0.11	1.06 (121)	0.29
Mother Education Level			
No formal education/Primary Level/Secondary	Ref		
Tertiary Level	-0.08	-0.75 (121)	0.45
Father Occupation			
Pensioners/Unemployed, Technicians and support services	Ref		
Manager and professionals	-0.08	-0.74 (121)	0.46
Mother Occupation			
Pensioners/Unemployed, Technicians and support services	Ref		
Manager and professionals	0.06	0.58 (121)	0.56
Number of Siblings	0.02	0.60 (121)	0.55
Locality of Origin			
Rural	Ref		
Urban	0.13	0.96 (121)	0.34
Personality			
Extraversion	0.32	4.04 (121)	**0.00**
Agreeableness	0.51	5.68 (121)	**0.00**
Conscientiousness	0.76	8.24 (121)	**0.00**
Neuroticism	-0.27	-3.99 (121)	**0.00**
Openness	0.32	2.64 (121)	**0.01**

Ref = reference group

Table 4 shows the predictors of grit among the medical students after adjusting for the confounders. The multiple linear regression analysis showed that three predictors were significantly associated with grit, explaining 47% of the variance (R^2^ = 0.47). All assumptions (linearity, independence, normality of the response variable, homoscedasticity, and fit of independent numerical variable) for multiple linear regression were met. An increase in the mean extraversion score by 1, b = 0.20 (95% CI: 0.07–0.32) would increase the mean grit score by 0.20. Similarly, a student with an increase in the mean agreeableness score by 1, b = 0.28 (95% CI: 0.12–0.44), would have an increase in grit score by 0.28, while an increased in the mean conscientiousness score by 1, b = 0.6 (95% CI: 0.42–0.77) would result in an increase in grit score by 0.6 points, independently.

**Table 4:** Multiple linear regression (MLR) on the predictors of grit among medical students

Variables	Grit				
	b	SE	T statistics	95% CI	*P* value
Agreeableness	0.28	0.08	3.53	(0.12–0.44)	0.01
Extraversion	0.20	0.06	3.08	(0.07–0.32)	0.00
Conscientiousness	0.60	0.09	6.6	(0.42–0.77)	0.00

MLR stepwise method

Variables included in the model are race, extraversion, agreeableness, conscientiousness, neuroticism, and openness

The model assumptions were met, with no multicollinearity or interactions between independent variables, R^2^ = 0.47

## Discussion

This study aimed to determine the level of grit and its association with sociodemographic, educational characteristics, and personality traits of the undergraduate medical students at this university. The mean grit score of the undergraduate medical students in this university was 3.43, which is comparable to their counterparts in Pakistan (3.22) and Singapore (3.60) but lower than those in the United States (US) (4.01) [4, 7, 29]. The medical students in the US were older, with a mean age of 27 years old [4], whereas the median age for students in this study was 23. The higher grit score among medical students in the US could be explained by previous literature, which has consistently shown a positive relationship between age and grit [3, 13, 18, 30]. However, our study could not show a significant association between age and grit, likely due to the homogenous nature of our population, which has a median age of 23 (IQR 2). Based on regression analysis, our study also found that other sociodemographic factors, such as ethnicity, were not significant predictors of grit. This finding was shared by a systematic review by Fernández-Martin et al., which also showed no significant difference in grit scores according to a person’s ethnicity [13].

Regarding the relationship between grit and personality traits, our study showed that conscientiousness predicted grit. Duckworth et al. looked at the relationships between grit and the Big Five personality traits and discovered a strong correlation with conscientiousness [3]. A meta-analysis also concluded that conscientiousness strongly correlates with grit [30]. Some literature argued that grit and conscientiousness were similar attributes [31], but Duckworth et al. disagreed [3]. While conscientiousness was described as being hardworking, responsible, self-disciplined, and thorough [20], this facet did not capture the “passion and perseverance” characteristics of grit, specifically the adherence and persistence to reach long-term goals [3].

Another finding of this study was the positive association between grit and the extraversion personality, implying that medical students who are sociable and extroverted are likely to exhibit grit. Duckworth et al. and Eskreis et al. also reported that extraversion was positively correlated with grit [3, 9]. A student who scores high on the extraversion personality tends to be confident and affectionate, which can be positive attributes for medical students. They can conduct themselves with confidence while at the same demonstrating care and devotion towards their patients. On the other hand, extroverted students are also fun-loving and tend to seek out time to socialise, which can be detrimental to their studies if done excessively. Thus, personalised coaching and monitoring with an academic advisor is crucial to ensure these students stay focused despite their extraversion traits. In contrast, Lin et al. conducted multiple linear regression to investigate the relationship between personality traits and grit. They found that extraversion did not significantly predict grit among Taiwanese high school students [32]. The findings from these studies suggested that extraversion personality is significantly associated with grit among undergraduate students, including medical students, but not among high school students [3, 9, 32]. High school lessons are more structured and often conducted in classroom-style learning; thus, having an extraversion trait may not significantly affect their grit. In the university, however, various teaching methods are employed and require higher cognitive ability; thus, students with higher extraversion traits need to exhibit a higher level of grit to stay on track in their studies. Nevertheless, these mixed findings add to the body of knowledge and should be explored more in future research.

This study also found that the agreeableness personality was positively associated with grit. It shows that medical students who are more cooperative and empathetic have a higher level of grit. Agreeable medical students show more concern for others than disagreeable students. Thus, they persevere to complete projects and tasks despite challenges so as not to cause harm or inconvenience to others. This finding was also supported by Lin and Chang, who found that an agreeableness personality predicted higher grit scores among Taiwanese high school students [32].

Our study discovered that prior academic achievements, such as the number of A grades in their high school certificate exam and their CGPA in pre-university preparatory courses, did not predict grit in this cohort of medical students. In the US, the Scholastic Aptitude Test (SAT) is an entrance exam commonly used by universities to decide on admissions [33]. Duckworth found that undergraduate students with lower SAT scores had higher grit levels [3]. The conflicting findings from our study may be explained by the population comprising high-achieving medical students. Almost all of them scored a perfect CGPA (4.0). Cognitive abilities were not a significant predictor of grit among these students. In the context of high-performing individuals, other factors, such as learning styles and self-efficacy, need to be explored.

### Strengths, limitations and implications for practice and future research

This study’s strength includes using a well-validated questionnaire to measure grit in the study population. Furthermore, the statistical analysis with multiple linear regression demonstrated the independent association between the predictors and grit while controlling for other confounders that may also influence grit. Next, to the best of our knowledge, this is the first study assessing the association between personality, sociodemographic, and academic characteristics with grit among medical students in Malaysia.

This study has some limitations. First, this cross-sectional study only established the association between the predictive variables and grit. This study design would only show the relationship between variables but not causality; thus, the results should only be interpreted in this context. Secondly, the participants recruited for this study were from a single medical institution, which consists of Malays as the majority ethnic group. Hence, the association between grit and other major ethnicities in Malaysia, such as Chinese and Indian, could not be explored. As a result, the findings from this study may not be generalisable to other medical schools with diverse ethnic groups. Future studies should include medical students from other universities to enable generalisation of the findings. Thirdly, data collection for this study was conducted via an online questionnaire. The response rate was low, a common issue in online data-gathering compared to face-to-face methods. This non-response bias may affect generalisation to the medical student population in this university; however, this bias is minimised due to the homogenous nature of our samples. Lastly, this study relied on results from the self-administered questionnaire; thus, the results may be subject to self-report bias. Lastly, data were collected during the COVID-19 pandemic. The findings from the literature suggest that the pandemic may influence the grit scores among students, and thus, the results should be interpreted with care. Most of the world’s population, including medical students, were subjected to unprecedented adversities, such as lockdowns and various health hazards [34]. A study reported that university students experienced fear and worry about their health, increased concerns related to their academic performance and reduced social interactions due to physical distancing [35]. Another study showed that during this pandemic, the negative impact of loneliness on academic stress was reduced by grit [36]. This study also suggested that as grit is a dynamic trait, it could be taught to students to build resilience and prevent academic stress during these difficult times [36].

Understanding the association of the significant variables with grit has important implications for medical education. It enables targeted and specific interventions for medical students with low grit levels. This study demonstrated that students with lower conscientiousness, agreeableness and extraversion traits would have lower grit. Grit can be enhanced through external factors [12]. Thus, individualised coaching and role-modelling initiatives, such as a mentor-mentee program, would improve grit among medical students and ensure that the students are supported in achieving satisfactory academic outcomes and subsequently finishing their medical course.

Regarding future research implications, other factors that may affect grit, such as self-control, self-efficacy, and general intelligence, should also be included in the regression analysis. The relationship between these students’ grit scores and academic performance, such as examination results and total duration to complete their medical course, could also be studied.

## Conclusions

The grit level among medical students in this university was comparable to that of other Asian medical students. Conscientiousness, agreeableness, and extraversion were associated with higher grit. As grit is a dynamic trait, appropriate interventions should be implemented to foster and increase it among these students.

## Data Availability

The datasets are stored at the Department of Primary Care Medicine, Faculty of Medicine, Universiti Teknologi MARA, Malaysia. Data may be shared upon reasonable request to the corresponding author and subjected to data protection regulations.

## Abbreviations

IQ: Intelligence quotient
GPA: Grade point average
Grit-S: Short Grit Scale
COI: Consistency of interest
POE: Perseverance of effort
BFI: Big Five Inventory
COVID-19: Coronavirus Disease 2019
MUET: Malaysian University English Test
MLR: Multiple linear regressions
SD: Standard deviation
IQR: Interquartile range
CI: Confidence intervals
